# Dreams

**Published:** 1886-11

**Authors:** 


### DREAMS.

(*Continued.*)

The realm of dreams is eminently spiritual. Time and space have no share in its mysteries, and oppose no obstacle to the completeness of its marvelous manifestations. A dream of a single moment may comprise many months or even a lifetime.

The eminent Dugal Stewart ascribed this to the fact that in dreaming the images created by our imagination appear to be realities, whereas those which we form when awake are known to us to be mere fictions, and hence not subject to the laws of time. Notwithstanding this explanation has been adopted by De Vere, in his elaborate work entitled "Modern Magic," it does not seem to us as affording a satisfactory explanation of this peculiar phase of the phenomonon.

The main difficulty in the way of presenting any comparative explanation of the nature and philosophy of dreams as related to our intellectual exertions in a normal state of wakefulness, lies in the utter dissonancy of the two states or conditions under which they seperately occur. It is within the experience of every dreamer, that oftentimes the thread and narrative of his dream, run on in an unbroken chain of events, covering apparently long periods of time, the minute circumstances and detail of which, to recount consecutively, or even to think out, would consume no inconsiderable time. On the other hand, the images created by us when awake and "known to us to be mere fictions," are not independent of time and space, even in thought, hence they compare with dreams only by contrast, there being in reality, little or no resemblance the one with the other.

Again certain actualities of sound, confusion and violence are not unfrequently incorporated in our dreams, as if in natural course, thus forming or assisting to form, a series of unbroken links in a lengthy chain of adventure or fantasy.

Robert Dale Owen furnishes us with an example in point. It was during his official sojourn in Naples that he dreamed of seeing several houses on fire, differently located, from which he argued in his sleep that it was the work of an incendiary. Following this were several distinct reports which he ascribed to a civil uprising against the Government and awaking at this point he became aware "that some persons were letting off fire crackers in the street."

The same evening there had been an alarm of fire in his vicinity, to which Mr. Owen attributed the early portion of his dream, as it is well known that the more absorbing incidents of the day often furnish the subject of our dreams by night.

One of the clearest and best illustrations of the almost utter annihilation of time and space in dreams from incentive to conclusion, which has come under our observation, is that of a writer in the "National Magazine," upwards of thirty years ago. This writer says: "On a very warm afternoon I was sitting in a somewhat lazy posture, listening to a friend, who was reading the *Christian Advocate and Journal.* As he commenced the obituary of a deceased preacher, I became drowsy, and although I felt considerably interested in the article, soon fell asleep and dreamed.

I thought I was standing by the bedside of the sick man, watching the progress of his disease, while a number of anxious friends sat in different parts of the room, or hung quietly over the bed. In the course of what seemed to me five or six hours, death came and released the sufferer, amid the sobbings and prayers of afflicted relatives. I remained with the family a day or two, until the funeral. The assemblage on that occasion was large, and the services were long and impressive. The funeral sermon, which was preached in the house, appeared to me to be nearly an hour in length, I listened to it with great interest and shall never forget the solemn impression it made upon my mind. After this, a procession of carriages was formed, and the deceased preacher was borne a distance of ten or twelve miles to his grave. He was buried at the side of a large, plain, old-fashioned brick church which stood near the corner of two streets. Here the funeral service was read, and after seeing the grave filled up, the company slowly departed. I lingered behind to indulge in the serious reflections that had been excited in me by the mournful occasion. I very well recollect standing in front of the church, at some little distance, and remarking to myself that, in case a monument should be erected over the remains, it would not look well, unless there should also be one on the other side of the church, to correspond with it. After this reflection I turned to leave the spot, and suddenly awoke. You may judge of my surprise when I found my friend still reading the obituary, and that he had read but two lines of it during my sleep."

Had the narrator of this remarkable dream, made himself acquainted with the facts and circumstances of the deceased minister's funeral, it is not altogether unlikely that they would have proved to be the prototype of his dream. Equally strange things have happened, and are happening, every day.

In its quiescent state of receptivity, the brain may be likened to the photographers sensitive plate, whereon are wrought with the suddenness and brevity of the lightening's vivid flash, the life-like images which it retains, even as the memory holds its own, in time's advancing years.

The chronicles of past ages, both sacred and profane, are replete with accounts of dreams, and many ancient writers have made them the subject of lengthy disquisitions.

"In a dream, in a vision of the night, when deep sleep falleth upon men, in slumberings upon the bed; then He openeth the ears of men and sealeth their instruction," says the author of the book of Job, which is commonly accounted the earliest scriptural mention of dreams, followed by others equally significant, in both the old and new testaments, wherein both their inspirational and prophetic nature are solemnly attested. "The early Christians" says De Vere, "foreseeing martyrdom, very frequently received in dreams an intimation of their impending fate, under symbolic forms, and, what was quite peculiar to their visions was, that they often extended to the pagan jailors and keepers, whose minds had been excited by witnessing the sufferings and the constancy of their victims, and who in many cases, became in consequence of those dreams, converts to the new faith."

The same writer remarks that "Men of cool judgment and clear mind have at all times been found on the side of believers. Even our great Franklin, with his eminently practical mind, and well-known aversion to every kind of superstition, firmly trusted in views which he believed to have come to him in dreams."

Great stress is laid upon dreams by the ancients and the persons appointed to interpret them, were accorded a very distinguished rank in the State. Indeed, the chronicles of nearly all the nations of antiquity are not wanting in this species of lore, nor is the classic literature of the past, at all deficient in this respect. Virgil ascribed the source of idle dreams to a mischievous sprite that lurked in the entrance of the infernal regions.

"Full in the midst of this infernal road
An elm displays her dusky arms abroad:
The god of sleep there hides his heavy head;
And empty dreams on every leaf are spread."

Homer describes the avenues by which dreams find their way from the Elysian Fields to the upper world, but in rare instances they were said to be sent down from the throne of Jupiter, as in the case of Agamemnon, who was persuaded by a vision, to give battle to the Trojans without the aid of Achilles.

The *manes*, or ghosts of the dead, says Tibullus, a Roman poet, were believed to send pleasant dreams, with salutary admonitions respecting futurity, to their former friends on earth, hence "it became a principle part of domestic worship to appease them,"

"Lest the neglected *manes* sad dreams send."

The ceremony used for this purpose was the offering of a cake sprinkled with salt. In another passage this poet describes himself as occupied in expelling evil dreams from the slumbers of his sick mistress.

"The thrice-blest cake have I prepared to keep
From sad tumultous dreams her sacred sleep."

Milton who believed in the influences of good and evil spirits, upon mortals, represents Satan as inspiring evil dreams in Eve.

"Assaying by his dev'lish art to reach
The organs of her fancy, and with them forge
Illusions as he list, phantasms, and dreams —"

Again, Adam is made to assert,

"Millions of spiritual creatures walk the earth,
Unseen, both when we wake and when we sleep."

We might pursue this subject, so replete in illustration and incident, indefinitely, but mindful that the patience of the readers has already been overtaxed, we will close with a single quotation from the great master genius of poetry, wherein he makes "Queen Mab" play the part of a mischievous elf, who

"Gallops night by night
Through lover's brains, and then they dream of love."
* * * * * * *
"And sometimes comes she with a tithe-pig's tail,
Tickling the parson as he lays asleep;
Then dreams he of another benefice."

We have purposely omitted the particulars of any of those more remarkable discoveries, forewarnings and presentiments which are known to have been given in dreams, but it is our purpose to call attention to them in a separate article.

